# Genetic Variation, Structure, and Gene Flow in a Sloth Bear (*Melursus ursinus*) Meta-Population in the Satpura-Maikal Landscape of Central India

**DOI:** 10.1371/journal.pone.0123384

**Published:** 2015-05-06

**Authors:** Trishna Dutta, Sandeep Sharma, Jesús E. Maldonado, Hemendra Singh Panwar, John Seidensticker

**Affiliations:** 1 Smithsonian Conservation Biology Institute, National Zoological Park, Washington, DC, United States of America; 2 Division of Mammals, National Museum of Natural History, Smithsonian Institution, Washington, DC, United States of America; 3 Peace Institute Charitable Trust, Mayur Vihar, Delhi, India; University of British Columbia Okanagan, CANADA

## Abstract

Sloth bears (*Melursus ursinus*) are endemic to the Indian subcontinent. As a result of continued habitat loss and degradation over the past century, sloth bear populations have been in steady decline and now exist only in isolated or fragmented habitat across the entire range. We investigated the genetic connectivity of the sloth bear meta-population in five tiger reserves in the Satpura-Maikal landscape of central India. We used noninvasively collected fecal and hair samples to obtain genotypic information using a panel of seven polymorphic loci. Out of 194 field collected samples, we identified 55 individuals in this meta-population. We found that this meta-population has moderate genetic variation, and is subdivided into two genetic clusters. Further, we identified five first-generation migrants and signatures of contemporary gene flow. We found evidence of sloth bears in the corridor between the Kanha and Pench Tiger Reserves, and our results suggest that habitat connectivity and corridors play an important role in maintaining gene flow in this meta-population. These corridors face several anthropogenic and infrastructure development threats that have the potential to sever ongoing gene flow, if policies to protect them are not put into action immediately.

## Introduction

The importance of maintaining meta-populations of large carnivores in a landscape with habitat connectivity between source populations is an intuitive and logical concept supported by a large body of scientific evidence [1–3]. Small isolated populations that lack connectivity are at risk of suffering from low genetic variation, which combined with other factors, reduces the probability of overall persistence of populations and species [4,5]. Large carnivore conservation is a daunting task in human-dominated landscapes, particularly those not connected by functional linkages or a permeable matrix between natural areas [6]. Carnivores move over long distances, have high dispersal capabilities, and need corridors for movement, dispersal, and gene flow [2,7–9].

The sloth bear (*Melursus ursinus*) is a large wide-ranging carnivore species endemic to the Indian subcontinent (S1 Fig). It is a medium-sized ursid (adult males 75–140 kg; females 55–95 kg) and the only bear species with a suite of specialized adaptations for myrmecophagy (diet consisting of ants and termites) [10]. Sloth bears are omnivorous [11,12], feeding primarily on social insects (ants and termites) and a broad spectrum of plant material such as leaves, shoots, tubers, and seasonal fruits and flowers [13,14]. Their diet strongly follows the phenology of flowering and fruiting of plant species in their habitat. These resources are also collected and used by humans, which often lead to incidents of human-bear conflict [12,14,15].

Sloth bears are still widely distributed in a large part of their range in India, where patches of tropical forests still exist. The Western Ghats and Central India are major strongholds of their distribution in terms of population abundance and habitat availability [13]. In the recent country-wide census of large carnivores and their prey species [16], sloth bears were found to have the most widely recorded distribution of any large carnivore in the Central India and Eastern Ghats landscapes. Central India has the largest extent of habitat and largest population size of sloth bears in India, with ~180,000 km^2^ of sloth bear occupied forests [17]. However, their distribution has gradually decreased and become patchy due to habitat loss and fragmentation [13,18,19]. Within the current range of sloth bears, human activities are the predominant factors determining areas of occupancy [19]. Small isolated patches of forest and high human and road density are correlated with higher risk of sloth bear extirpation [13,20]. The continuous developmental and infrastructural growth (mines, roads, and dams) to support the growing economy of the subcontinent poses a great risk of further fragmentation and insularization of sloth bear occupied forests [19].

Bear policy documents such as the IUCN Bear Action Plan [21] and the National Bear Conservation and Welfare Action Plan (NBCWAP) of India [17] recognize that reducing the impacts of habitat fragmentation and human activities on sloth bear habitats is critical to sloth bear persistence, and also recommend research on the species at a landscape scale. The IUCN Bear Action Plan [21] emphasizes delineating discrete population units, rather than individual reserves, as the basis for their management and conservation strategies. These include establishing and managing additional protected areas and interconnecting and safeguarding corridors and buffer zones between protected areas. The NBCWAP [17] highlights the paucity of research and basic scientific information on sloth bears in India, and recommends genetic studies for recording presence/absence of bears in unsampled and non-protected areas.

Multiple studies have been conducted to understand population genetics of other bear species [22–28], but no information is available on population and landscape genetics of sloth bears. We undertook a project to fill existing gaps in information on sloth bear ecology, by describing the genetic variation in sloth bear populations in a large landscape consisting of four sloth bear populations in five tiger reserves interconnected by corridors in Central India. Given their generalist diet, patchy occupancy, and lack of information about their dispersal range and pattern, an ecological question of strong conservation relevance is how sloth bear allelic diversity is distributed within the landscape, and how existing corridors affect this allelic distribution. Our specific objectives in this study were to a) determine if sloth bears use corridors, b) describe the genetic variation and genetic structure of sloth bear populations in this landscape, and c) quantify gene flow among sloth bear populations in this landscape.

### Study Landscape

**Fig 1:**
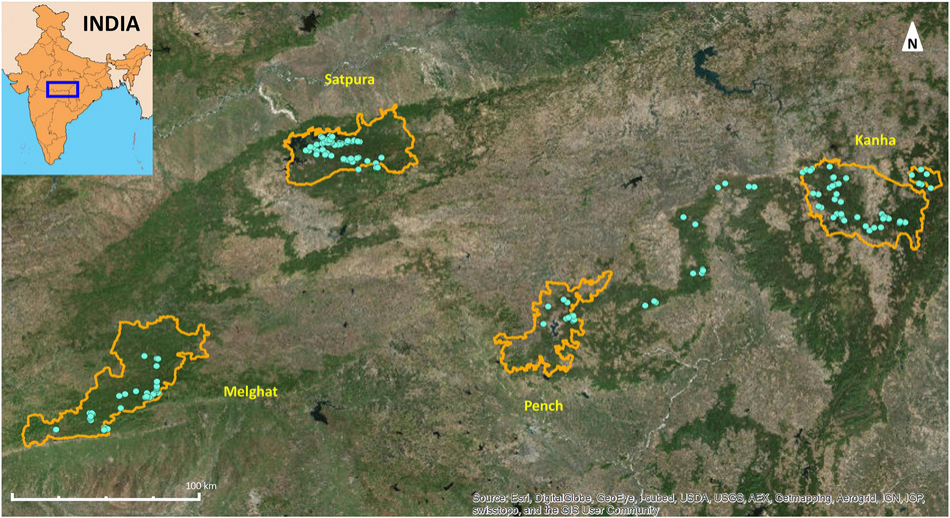
Map of the study landscape with locations of all sloth bear fecal and hair samples collected shown in blue dots. Tiger Reserve boundaries are indicated in orange.

We conducted our study in the Satpura-Maikal landscape in Central India (Fig 1). This landscape is characterized by the Satpura Range in the north and the Maikal Range in the east which form the catchments for the Narmada and the Tapti rivers and their tributaries. The landscape comprises five tiger reserves in two states: Kanha Tiger Reserve, Bori-Satpura Tiger Reserve (Satpura), and Pench Tiger Reserve are in the state of Madhya Pradesh (MP), while part of Pench Tiger Reserve and the Melghat Tiger Reserve are in the state of Maharashtra (Mh). Kanha and Pench are connected by a corridor and are located toward the eastern part of the landscape. Melghat and Satpura are connected by a corridor and lie to the west of the landscape. The connectivity between Kanha–Pench and Satpura–Melghat tiger reserves is largely contiguous forest cover, whereas the connectivity between Pench–Melghat and Pench–Satpura tiger reserves is fragmented. The intervening matrix in this landscape is composed of agricultural land and fragmented forest patches, interspersed with numerous small villages and towns.

## Methods

The office of the Principal Chief Conservator of Forests, Madhya Pradesh and Maharashtra granted permits to carry out this research. The same permissions applied to the Protected Areas as well as the Territorial Divisions which covered the corridors. We used noninvasively collected fecal and hair samples for this study. We did not capture or handle any animal, dead or alive.

### Field sampling

We conducted the field work for this study in 2009–2010 and used stratified random sampling to survey the landscape. We divided the entire landscape into 10 km^2^ grids, and carried out extensive surveys, covering 15000 km of forest trails during the field sampling. We collected sloth bear scat along forest trails and hair samples from trees that had sloth bear claw marks, and recorded the GPS location of each collection site. We stored samples in 100% ethanol until further analysis.

### Genetic analysis

We isolated genomic DNA using Qiagen’s QIAamp mini-stool kit for fecal samples and the QIAamp blood and tissue kit for hair samples following the manufacturer’s protocol (QIAGEN, Valencia, CA, USA). We used a previously optimized panel of seven microsatellite loci [29] that had high polymorphism, low error rates and had proved reliable for noninvasive genetic monitoring of sloth bears.

In order to improve amplification success, we used a two-step PCR amplification procedure [30]. In the pre-amplification step, we amplified all loci simultaneously. In the second PCR, we amplified individual loci using the product from the pre-amplification step as the template. The details of the PCR reactions are as follows.

Pre-amplification PCR: this PCR consisted of 25 μl reactions containing 0.5 U Ampli Taq Gold DNA Polymerase (Applied Biosystems, Carlsbad, California, USA), 0.01 μM each fluorescently-labeled forward primer, 0.01 μM each unlabeled reverse primer, 2 mM MgCl_2_, 1X Gold buffer, 0.1 mM of each dNTP, 2 μl 10X BSA, and 4–5 μl of the DNA extract. PCR conditions were: initial denaturation step (95°C for 10 min), 25 cycles of denaturation (95°C for 30 sec), annealing for 30 sec (60°C for brown bear primers and 50°C for the remaining primers in the pre-amplification step), and extension (72°C for 1 min), followed by a final extension step (72°C for 7 min). The second PCR: this locus-specific PCR consisted of 10 μL reactions which contained 0.5 U Ampli Taq Gold DNA Polymerase, 2 mM MgCl_2_, 1X Gold buffer, 0.1 mM of each dNTP, 2 μL 10X BSA, 0.2 μM of both forward and reverse primer and 1.5 μl of pre-amplified product as the DNA template. The PCR conditions for this locus-specific reaction were same as the pre-amplification, except that the annealing temperature was specific for each primer, and we amplified the product in 45 cycles instead of 25 cycles. We ran all PCR products on an ABI 3730xl sequencer, using GeneScan–500 LIZ (Applied Biosystems) size standard. We scored alleles using GeneMapper-4.1 (Applied Biosystems).

In order to control DNA quality, we used a two-step approach to select good quality samples for downstream analysis. In the first step, we amplified all samples with three loci (Umar2, CXX203, and G10L). Samples that amplified for at least 2 loci were further amplified with the remaining of the final panel of 7 microsatellite loci. Samples that amplified for at least 5 (out of 7) loci were retained for further analysis.

We used several lab precautions and analytical approaches to minimize errors that are characteristic of fecal DNA. We extracted DNA samples in small batches with a negative control in each set, used filter tips, and conducted extraction in a dedicated room that contained no previous bear PCR products. We used a modified multi-tubes approach [31], wherein we replicated amplification four times, and looked for consensus among genotyping scores. We discarded samples that did not amplify in the pre-amplification step, and selected samples that amplified for at least 5 of the 7 microsatellite loci to control the quality of data [29]. We used MICRO-CHECKER-2.2.3 [32] to detect loci containing errors due to scoring or stuttering, and large allele dropout. We estimated PCR success and genotyping error rates using GIMLET 1.3.3 [33].

### Data Analysis

#### Genetic diversity

We measured genetic diversity by estimating the number of alleles per locus (Na), observed (Ho), and expected (He) heterozygosity in GIMLET. We conducted tests for deviations from Hardy-Weinberg equilibrium (HWE) and linkage disequilibrium (LD) in GENEPOP 4.0.10 [34] with a Bonferroni correction applied for multiple comparisons. We used CERVUS 3.0.3 [35] to identify individuals and GIMLET to obtain P_ID_ (probability of identity) and P_ID(sibs)_ values. P_ID_ and P_ID(sibs)_ values are commonly used measures to evaluate the power of the selected panel of loci to distinguish individuals [36].

#### Genetic differentiation

To measure population-level differentiation, we measured pairwise F_ST_ values and 95% confidence intervals in FSTAT [37]. We used two approaches to test for isolation by distance. We tested isolation by distance between pairwise F_ST_ and Euclidean geographic distances in GENEPOP. In addition, we conducted Mantel tests between each pair of individuals within each of the four tiger reserves in GenAlEx 6.5[38].

F_ST_ has been demonstrated to be a poor estimator of genetic differentiation that may have been caused by recent habitat fragmentation, because it has a lag time of about 200 generations [39,40]. Individual-based analysis offer a greater resolution to understand genetic effects of recent landscape changes [40]. We used one multivariate and two individual-based Bayesian analytical methods to determine the number of genetic populations of sloth bears in our study landscape. First, we used the program DAPC (Discriminant Analysis of Principal Components) using the ADEGENET [41] package in R. DAPC is a multivariate method to identify the number of genetic populations (referred as K). DAPC is a two-step process, which first transforms raw genetic data using principal components analysis (PCA) and then maximizes genetic differentiation between groups, without making several commonly required assumptions (e.g., HWE, LD) about the underlying genetic data. DAPC executes k-means, a clustering algorithm which finds a given K number of clusters that maximize variation between groups. To identify the optimal K, the algorithm k-means is run sequentially with increasing K and the optimal clustering solution is selected by Bayesian Information Criterion (BIC). BIC is typically indicated by an elbow in the curve of BIC as a function of K.

We used the Bayesian assignment program, STRUCTURE [42], to detect genetic clusters (K) appropriate to describe our data. This widely used approach assigns individuals into K clusters in a way that minimize deviations from HWE and LD within each cluster. The program uses a Markov Chain Monte Carlo (MCMC) procedure to estimate the posterior probability that the data fit the hypothesis of K clusters. The program also calculates the fractional membership of each individual in each cluster (q). We let K vary between one and ten using the admixture model with correlated allele frequencies [43] and conducted ten independent runs for each K value. We used 500,000 iterations as burn-in and based the estimations on 500,000 additional iterations. We used the delta K criteria [44] to determine the number of genetic clusters.

We also used a spatially-explicit Bayesian analyses in TESS 2.3.1 [45]. TESS uses a hidden Markov random field model to compute the proportion of individual genomes originating in K populations. The algorithm accounts for spatial connectivity and incorporates a decay of membership coefficient correlation with distance, which is a property similar to isolation by distance. We ran the program with a burn-in period of 50,000 cycles and based the estimations on 100,000 additional cycles. We increased the maximum number of clusters from 2–10 (10 replicates for each value). Ancestry of each individual in each genetic group was recorded using the q matrix, which is analogous to the q matrix in STRUCTURE and describes the proportion of an individual’s genotypic ancestry that can be attributed to each identified genetic group.

#### Gene flow and detection of migrants

We used the q-matrix from the STRUCTURE results to detect individuals of admixed ancestry, residents and migrants. We considered individuals with q values > 0.80 as residents of the area where they were sampled; those with q values from 0.2–0.8 as admixed individuals that could not be readily assigned as residents or migrants; and those with q values <0.2 as migrants [46].

We used GENECLASS2 [47] to examine whether individuals were residents or migrants in the area from which they were sampled. We employed the Bayesian approach [48] to assign or exclude individuals to their sampling population. The latter approach was used along with the Likelihood computed from the population where the individual was sampled (L = L_home/L_max), as this does not assume that all potential source populations have been sampled. All tests were based on assignment criteria computed from 10,000 simulated populations of the same size as the sample populations.

Finally, we used BAYESASS1.3[49] to estimate recent gene flow (1–3 generations before present) between pairs of populations. BAYESASS is a Bayesian method with MCMC that measures gene flow between populations by identifying population-specific inbreeding coefficients and genotypic disequilibrium. It allows deviations from Hardy–Weinberg expectations within populations, and assumes linkage equilibrium and that populations have not been subjected to genetic drift within the past 2–3 generations before sampling. The program uses allelic data to estimate the fraction of individuals that are residents (non-migration) and the fraction of individuals in one population that are migrants from another population (migration). We changed the number of delta values of proposed allele frequency, inbreeding (F) value, and migration rate changes to obtain the recommended 40–60% acceptance rates. Once the runs converged within the acceptance rates, we ran three independent runs to check for consistency in results. We used 3,000,000 iterations, of which 1,000,000 were burn in, and the sampling frequency was 2000.

To test the hypothesis about correlation of forest connectivity and genetic differentiation, we calculated least-cost distance and Euclidian distance using the program Linkage Mapper [50]. We used a single layer of forest cover as a predictor of genetic differentiation. We assigned lower cost to forest cover and higher cost to non-forested areas under the assumption that sloth bears would encounter higher costs when moving through non-forested areas. We performed Mantel test in the program PASSaGE [51] with 999 randomization to assess correlation between least-cost distance and Euclidian distance respectively with F_ST_ (a measure of genetic differentiation).

## Results

Over a period of two years, we collected 190 fecal and 4 hair samples from wild sloth bears throughout the Satpura-Maikal landscape (Fig 1). This includes fifteen samples collected from the Kanha-Pench corridor. We did not find any sloth bear samples in other corridors in the landscape.

We did not detect any evidence of stuttering errors and large allele dropout in any of the 7 microsatellite loci using MICROCHECKER. Genotyping error rates (dropout and false allele) were low (mean false allele: 0.0002; mean dropout: 0.05) (S1 Table). Two loci in Melghat population (G1A, and UarMU26) deviated from HWE, while all loci from remaining 3 TRs were in HWE. None of the 84 pairwise comparisons between loci across populations was in significant LD.

**Fig 2:**
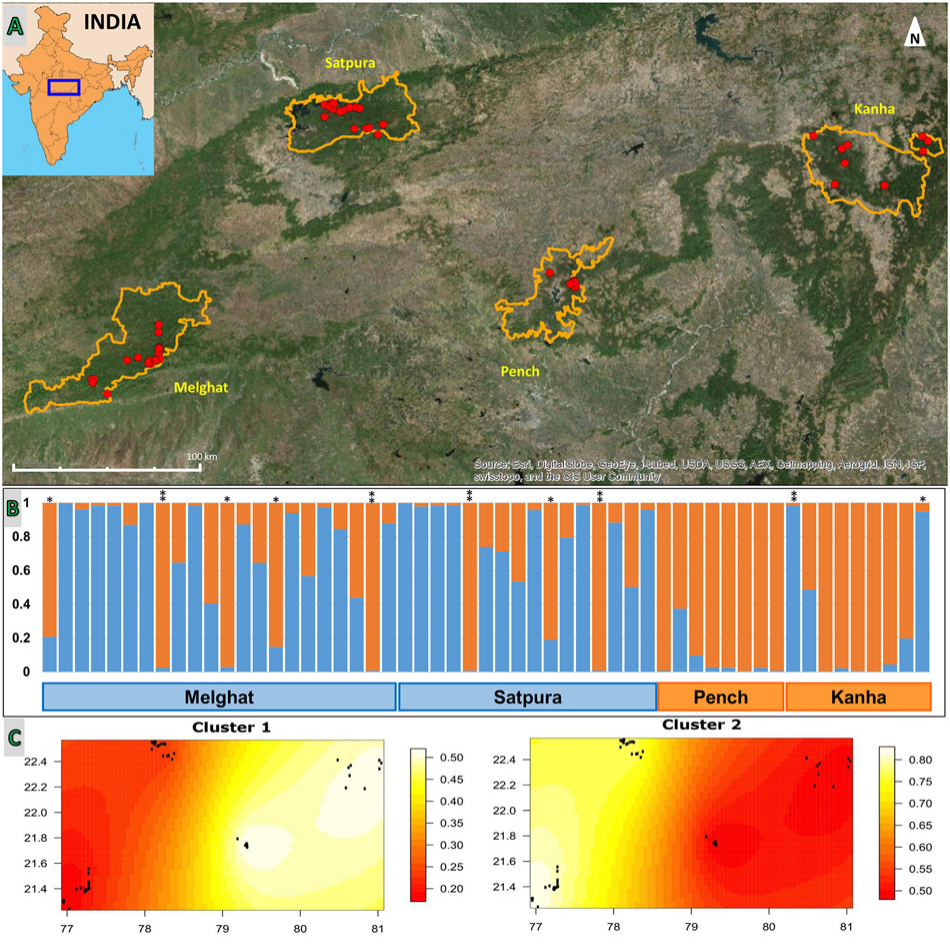
Results from genetic analysis of sloth bear fecal samples. (a) Map of study landscape with 55 individually identified sloth bear locations represented as red dots. Tiger Reserve boundaries are indicated in orange. (b) Bar plots from STRUCTURE show the assignment probabilities for each bear to either of the two genetic clusters. The y-axis shows the calculated membership coefficient (q). (c) Spatial interpolation of admixture proportions from analysis in TESS. Individuals are shown in black dots, x and y axis indicate latitude and longitude respectively, and membership is indicated by the color ramp. All individuals with a single star on the top were identified as potential migrants in STRUCTURE, and individuals with two stars were identified as migrants in both STRUCTURE and GENECLASS.

When we applied our quality control criteria, 89 samples out of the total 194 amplified for at least two of the three loci (Umar2, CXX203, and G10L). Fifty eight samples were retained for further analysis when we applied the criteria of amplification of at least 5 out of the total 7 loci. We were able to successfully amplify 80.78% of the total expected alleles. We identified 55 individuals (P_ID sibs_ 2.15E-03) from this pool, and there were three individuals that were recaptured twice using exact match criteria. The sample sizes across reserves were: 9 individuals from Kanha, 8 from Pench, 16 from Satpura, and 22 from Melghat (Fig 2a).

### Genetic differentiation

After controlling for sample size, allelic and private allele richness was comparable across the four tiger reserves (Table 1). The mean F_ST_ value of 0.042 was significant (95% CI 0.023, 0.065). Pairwise F_ST_ values were significant between Kanha and Satpura, Pench and Satpura, and Pench and Melghat (Table 2), which are all pairs of populations that have little to no structural connectivity between them. We did not detect any significant isolation by distance within any of the four tiger reserves (Kanha p = 0.23, Pench p = 0.06, Satpura p = 0.19, Melghat p = 0.46), or between pairs of tiger reserves (p = 0.50).

**Table 1:** Summary of genetic diversity in sloth bears from central India.

Population		Kanha (n = 9)					Pench (n = 8)					Satpura (n = 16)					Melghat (n = 22)				
Loci	Total no. alleles	Na	Ar	Pr	He	Ho	Na	Ar	Pr	He	Ho	Na	Ar	Pr	He	Ho	Na	Ar	Pr	He	Ho
CXX203	12	7	3.40	0.84	0.82	0.67	6	3.02	1.07	0.74	0.75	9	3.33	0.97	0.85	0.75	9	3.19	0.80	0.82	0.55
G10B	4	4	2.26	0.62	0.57	0.44	1	1.00	0.00	-	-	3	1.81	0.23	0.37	0.25	4	2.04	0.56	0.47	0.32
G10J	12	5	2.90	0.73	0.72	0.33	8	3.64	1.74	0.86	0.5	10	3.44	1.41	0.86	0.69	9	3.24	1.10	0.83	0.64
G10L	7	6	3.01	0.71	0.76	0.89	5	2.70	0.71	0.69	1	4	2.22	0.09	0.58	0.81	6	2.46	0.34	0.65	0.91
G1A	11	6	3.31	1.08	0.81	0.33	6	3.18	0.97	0.78	0.5	8	2.75	0.79	0.71	0.56	6	2.09	0.48	0.48	0.23
UarMu26	7	5	2.84	0.69	0.73	0.78	4	2.00	0.44	0.42	0.25	6	2.34	0.37	0.57	0.38	5	2.57	0.46	0.68	0.27
Umar2	9	7	3.25	1.22	0.81	0.89	7	3.27	0.92	0.79	0.63	4	2.23	0.23	0.56	0.44	5	2.53	0.59	0.65	0.55
Average		5.71	3.00	0.84	0.75	0.62	5.29	2.69	0.84	0.61	0.52	6.29	2.59	0.59	0.64	0.55	6.29	2.59	0.62	0.65	0.49

Na-Total number of alleles, Ar-Allelic richness, Pr-Private allele richness, He-Expected heterozygosity, Ho-Observed heterozygosity.

**Table 2:** F_ST_ values between populations.

	Kanha	Pench	Satpura	Melghat
Kanha	——	110/123	200/211	312/345
Pench	0.033 (-0.014,0.089)	——	95/123	163/173
Satpura	**0.037 (0.007,0.066)**	**0.086 (0.020, 0.185)**	**——**	85/118
Melghat	0.061 (-0.001, 0.141)	**0.059 (0.030, 0.087)**	0.009 (-0.005, 0.028)	——

Pairs of populations with significant F_ST_ values are in bold. Values in parenthesis indicate the 95% CI. Values above diagonals indicate Euclidean Distance and Least Cost Path Distance in kilometers (EucDist/LCPDist).

**Fig 3:**
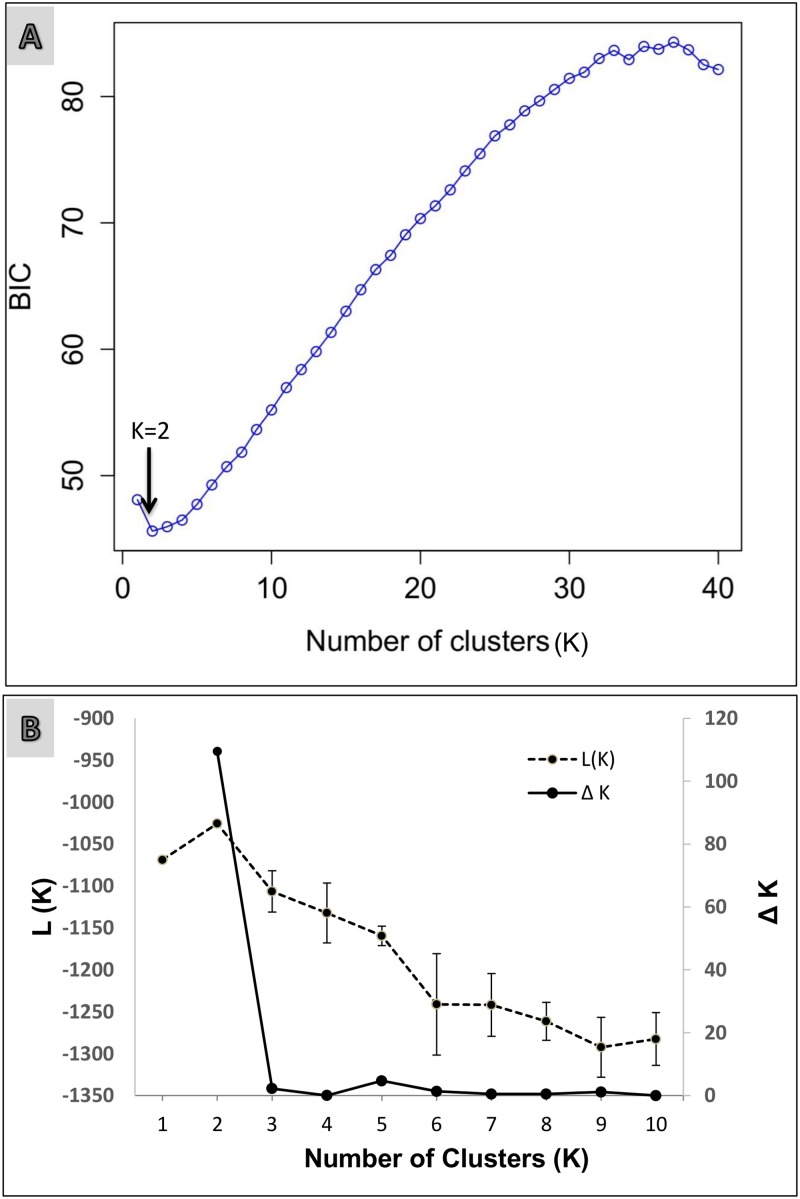
Clustering results from DAPC and STRUCTURE. (a) BIC plot from DAPC showing an elbow with an arrow at K = 2 (b) Mean Likelihood L(K) and standard deviations for K = 1–10 (dashed line) clusters over 10 independent runs and the estimate of ΔK using the Evanno et al. (2006) [44] approach (solid line).

The optimal number of clusters using three different approaches was K = 2 (Fig 3). In DAPC, the elbow in the curve of BIC was at K = 2 (Fig 3a). In the STRUCTURE analyses, the mode of the ΔK for all values of K from 1 to 10 was at K = 2 (Fig 3b). Over 10 runs at K = 2 [L(K) = -1025.52; St Dev of L(K) = 1.136], most individuals sampled in Kanha and Pench were assigned to one genetic cluster, whereas most individuals from Satpura and Melghat were assigned to another genetic cluster (Fig 2b). Using the spatially explicit clustering analysis in TESS, we detected two genetic clusters (Fig 2c), and the results of individual admixture were very similar to those from STRUCTURE.

To address the issue of the effect of asymmetric sample size (ranging from 8 individuals from Pench to 22 individuals from Melghat) on STRUCTURE results, we randomly reduced the number of individuals from Satpura and Melghat to make sample sizes similar across all four populations. In this analysis, we had the following sample sizes: Kanha (9), Pench (8), Satpura (9) and Melghat (8). We found that even after randomly removing individuals from Satpura and Melghat, we still detected K = 2 (S2 Fig). This additional test shows that our results are robust to differences in sample size.

### Gene flow and detection of migrants

The analysis of q-matrix of STRUCTURE results revealed that eleven individuals (20%) were admixed (one each in Kanha and Pench, four in Satpura and five in Melghat), ten individuals (18%) were potential migrants, and 34 individuals (62%) were residents. GENECLASS detected five of these potential migrant individuals as first-generation migrants. One individual sampled in Kanha (assigned to Satpura), two individuals in Satpura (assigned to Kanha and Pench), and two individuals in Melghat (assigned to Satpura and Pench) were identified as being migrants (Fig 2c).

In the BAYESASS analysis, the highest and lowest proportions of non-migrants (both residents and admixed) were detected in Melghat (0.95, 0.8717–0.9984) and Satpura (0.70, 0.6678–0.7635), respectively. The highest proportion of migrants were from Melghat to Satpura (0.26) and the lowest proportion of migrants were from Pench to Melghat (0.01) (Table 3).

**Table 3:** Contemporary migration rates between sampled populations.

	Kanha	Pench	Satpura	Melghat
Kanha	***0*.*74***	0.08	0.02	0.01
	(0.6697, 0.8971)	(0.0007, 0.2352)	(0.0002, 0.0869)	(.000, 0.059)
Pench	0.07	***0*.*77***	0.02	0.01
	(0.0008, 0.2035)	(0.6696, 0.9707)	(0.0001, 0.0746)	(.0001, 0.0648)
Satpura	0.07	0.06	***0*.*70***	0.02
	(0.0009, 0.2061)	(0.0003, 0.2326)	(0.6679, 0.7629)	(.0001, 0.0938)
Melghat	0.12	0.10	0.26	***0*.*95***
	(0.0253, 0.2450)	(0.0014, 0.2433)	(0.1756, 0.3167)	(0.8642, 0.9982)

Migration rates from a source population are in rows while columns indicate migration rates into a population. Diagonal numbers in bold italics indicate non-migration rates and values in parenthesis indicate the 95% confidence intervals.

To assess whether genetic differentiation and forest cover are correlated, we built resistance layers using a layer of forest cover and calculated Euclidian and least cost path distances. We found that neither Euclidian distance (R = 0.2404, p = 0.53), nor least cost distance (R = 0.2422, p = 0.51) was significantly correlated with F_ST_.

## Discussion

This is the first study of population and conservation genetics of sloth bears. We confirmed the reliability of a panel of seven polymorphic microsatellite loci to monitor sloth bear populations using noninvasively collected samples. We also demonstrate its utility to study population genetic parameters and gene flow between sloth bear source populations in the Satpura-Maikal landscape in Central India.

It has been documented that collecting fresh samples improves amplification success [52]. However, we collected fecal and hair samples opportunistically regardless of their age (fresh and old) in order to more extensively sample sloth bear presence throughout the landscape. We found fifteen sloth bear fecal samples in the Kanha-Pench corridor, confirming that sloth bears do indeed occupy and use this corridor. All the samples from the corridor were old when we collected them, and despite several attempts, we could not obtain reliable microsatellite data from these scats. In future studies, more effort to collect fresh scat from the corridors would help to identify the source populations of the individuals in the corridor.

Despite the challenges associated with amplification of noninvasive samples [53], we were able to detect 55 different individuals from 194 field collected samples and obtain reliable measures of allelic richness and heterozygosity. Our results show that sloth bears in this landscape have moderate levels of genetic diversity. After controlling for unequal sample sizes, bears in all tiger reserves had comparable levels of allelic richness.

Differences in study design and types and numbers of markers hinder direct comparisons between studies that have examined levels of genetic variation in different species of ursids. However, our results suggest that the sloth bear population of central India has moderate levels (Ho = 0.53, Na = 8.8) compared to estimates of population genetic diversity previously reported for Brown bears (*Ursus arctos*) (Ho = 0.298–0.853, Na = 2.13–8.312) [22, 24, 28], Polar bears (*Ursus maritimus*) (Ho = 0.68) [27] and American Black bears (*Ursus americanus*) (Ho = 0.287–0.800, Na = 2.25–9.5) [23, 54].

We used three different analytical approaches to detect genetic structure among sloth bear populations of Central India. These methods differ in their assumptions, and whether or not they include spatial information. All three analytical approaches indicate the presence of two genetic clusters. These two clusters are pairs of populations that are interconnected by existing corridors (Kanha-Pench and Satpura-Melghat). Previous studies on tigers (*Panthera tigris*) and leopards (*Panthera pardus*) in the Satpura-Maikal landscape [55–58] have established the functionality of these corridors and their importance in maintaining the historical levels of gene flow in this landscape. Findings from this study further bolster the evidence for the effectiveness of these corridor.

The analysis of migrants and contemporary gene flow indicate ongoing genetic exchange between the populations. For example, five individuals (9% of the identified individuals) were confirmed as first-generation migrants and 20% of the individuals were of mixed ancestry, indicating admixture between the populations. These results show that sloth bears in this landscape are moving from one population to another and effectively breeding in the recipient population, thus maintaining genetic exchange and preventing further genetic subdivision in this landscape.

We did not find a significant relation between forest cover and genetic distance. We think that this could be due to several factors: (1) Although least cost distances on an average are 21 km longer than Euclidian distances, the distances are highly correlated (R = 0.97 p = 0.04). The way this landscape is configured, it just so happens that Euclidian distance and least cost paths overlap to a certain degree. (2) Forest cover alone may not be a sufficient explanatory variable for genetic differentiation. Other factors such as food availability (ants, termites and fruiting plants), and impact of human density, transportation networks and other infrastructure would be critical and ecologically meaningful for dispersing sloth bears.

The best way to thoroughly establish the use and effectiveness of corridors by any species would be to combine genetic studies with movement tracking studies of multiple individuals to map their movement and effective dispersal through different landscape features. However, this is beyond the scope of our study, and our results using noninvasive genetic sampling provide strong evidence about the functionality of these corridors.

Fragmentation and loss of habitat is one of the most serious causes of population decline and species extinction globally [59], which also affects the genetic variation and viability of populations. A fragmented landscape presents a higher cost to individuals dispersing through such areas, and potentially decreases effective dispersal and genetic exchange. The Satpura-Maikal landscape has experienced tremendous habitat loss and fragmentation over large time scales that echo human occupation and activities in this region [58]. This landscape lost 76% of its forest cover to land conversion for agriculture and urbanization in the past 300 years (1700–2000) [58]. Despite this severe habitat loss, the sloth bear populations in this landscape have maintained moderate level of genetic diversity. However, several new threats are emerging that pose risks to this functional connectivity. Some of the very specific issues that need immediate intervention for the conservation of metapopulations of sloth bears and other large carnivores such as tigers, leopards, and dholes are:

1. Coal mining: The Central Indian Highlands are one of the major coal fields of India. There are several operational coal mines in Pench-Satpura corridor. In recent years, new mining leases were granted to mines proposed to be located inside this corridor. Mining activity has the potential to increase anthropogenic disturbance and reduce the connectivity through this important corridor [60].
2. Widening of National Highway 7 (NH7): NH7 runs parallel to the eastern boundary of Pench tiger reserves (MP and Mh) for a length of 65 km. A stretch of 8.9 km of this highway intersects the Kanha-Pench corridor close to Pench tiger reserve (MP). The proposed widening of NH7 [61,62] near these two tigers reserves from two lanes to four lanes will not only result in loss of forest cover and increased chances of collision-related mortality of wildlife but also sever the crucial linkage between Kanha and Pench. This road-widening project may impact animal movement and gene flow between these two source populations, as has been shown in other carnivores [63,64].
3. Gauge conversion of railway line: A project to convert a 74.9 km stretch (Nainpur to Balaghat) of narrow-gauge railway line to broad-gauge is in progress. A 17.98 km long section of this railway line passes through forested area in the Kanha-Pench corridor and would impact 69.75 hectares of forest land [61]. In addition to this forest loss, the elevated broad-gauge tracks, increased speed of trains on broad-gauge rail (from a maximum speed of 40 kmph to 100 kmph), and increased frequency of trains would diminish the functional value of this corridor, as has been shown for brown bears in Slovenia [65].
4. Teak-monoculture: Multiple forest blocks adjacent to the tiger reserves and in the corridors in the Satpura-Maikal landscape have been demarcated for teak production. These monoculture blocks are devoid of any understory or fruiting trees [66,67], and apart from offering cover, may not be used by sloth bears.

Linear infrastructure features such as roads and railway lines have been shown to have severe adverse impacts on the behavior, movement, and gene flow of wildlife [63,68]. For example, Riley et al. (2006) [64] evaluated movement and gene flow of bobcats (*Lynx rufus*) and coyotes (*Canis latrans*) across a California freeway using genetic methods and radio-telemetry and found that although a few individuals of both species moved across the road, this movement was not enough to compensate for the restricted levels of gene flow caused by the freeway. Kerley et al. (2002) [69] revealed that primary roads and associated human disturbance decrease the survivorship and reproductive success of tigers in the Russian Far East.

Some of these detrimental impacts can be mitigated by the use of specially designed structures such as overpasses and underpasses [70]. A few studies have demonstrated the efficacy of these structures in safeguarding animal movement, dispersal, and gene flow [71,72]. However, there may be species-specific responses to the same barrier. For example, Sawaya et al. (2013) [72] showed that although crossing structures provided genetic connectivity for both species, grizzly bears showed higher genetic discontinuity than black bears across a major transcontinental highway in Banff National Park, Canada. The usefulness of crossing structures has yet to be tested at any extensive scale in different eco-regions of the world [71].

We demonstrated that forest corridors in the Satpura-Maikal landscape are being used and are functional for movement and gene flow of sloth bears. However, no government policies are in place to safeguard these crucial areas of connectivity. Our study calls for immediate policy intervention for conservation planning to maintain linkages between protected areas in this landscape.

## Supporting Information

S1 Fig Range map of Sloth bear (*Melursus ursinus*) showing the study landscape. (DOCX)

S2 Fig Results from STRUCTURE analysis with fewer samples from Satpura and Melghat. (DOCX)

S1 Table Characteristics and error rates of genotyping 55 individual sloth bears from Satpura-Maikal landscape in India. (DOCX)

## References

[1] Harris LD. The fragmented forest: island biogeography theory and the preservation of biotic diversity. Chicago: University of Chicago Press; 1984.

[2] Carroll C, Noss RF, Paquet PC. Carnivores as focal species for conservation planning in the Rocky Mountain region. Ecol Appl. 2001;11: 961–980.

[3] Larkin JL, Maehr DS, Hoctor TS, Orlando MA, Whitney K. Landscape linkages and conservation planning for the black bear in west-central Florida. Anim Conserv. 2004;7: 23–34.

[4] Harris LD, Silva-Lopez G. Forest fragmentation and the conservation of biological diversity In: Fiedler PL, Jain SK, editors. Conservation biology. Boston, MA: Springer US; 1992 pp. 197–237.

[5] Saunders DA, Hobbs RJ, Margules CR. Biological consequences of ecosystem fragmentation: a review. Conserv Biol. 1991;5: 18–32.

[6] Crooks KR, Sanjayan M. Connectivity conservation. Cambridge, UK: Cambridge University Press; 2006.

[7] Proctor MF, McLellan BN, Strobeck C, Barclay RM. Genetic analysis reveals demographic fragmentation of grizzly bears yielding vulnerably small populations. Proc R Soc B Biol Sci. 2005;272: 2409–2416. 16243699 10.1098/rspb.2005.3246 PMC1559960

[8] Chetkiewicz CLB, St. Clair CC Boyce MS. Corridors for conservation: integrating pattern and process. Annu Rev Ecol Evol Syst. 2006; 317–342.

[9] Rabinowitz A, Zeller KA. A range-wide model of landscape connectivity and conservation for the jaguar (*Panthera onca*). Biol Conserv. 2010;143: 939–945.

[10] Sacco T, Van Valkenburgh B. Ecomorphological indicators of feeding behaviour in the bears (Carnivora: Ursidae). J Zool. 2004;263: 41–54.

[11] Gopal R. Ethological observations on the sloth bear (*Melursus ursinus*). Indian For. 1991;117: 915–920.

[12] Rajpurohit KS, Krausman PR. Human-sloth-bear conflicts in Madhya Pradesh, India. Wildl Soc Bull. 2000;393–399.

[13] Yoganand K, Rice CG, Johnsingh AJT, Seidensticker J. Is the sloth bear in India secure? A preliminary report on distribution, threats and conservation requirements. J Bombay Nat Hist Soc. 2006;103: 172.

[14] Bargali HS, Akhtar N, Chauhan NPS. Feeding ecology of sloth bears in a disturbed area in central India. Ursus. 2004;15: 212–217.

[15] Akhtar N, Singh Bargali HS, Chauhan NPS. Sloth bear habitat use in disturbed and unprotected areas of Madhya Pradesh, India. Ursus. 2004;15: 203–211.

[16] Jhala YV, Qureshi Q, Gopal R, Sinha PR. Status of tigers, co-predators and prey in India. National Tiger Conservation Authority, Govt. of India, New Delhi, and Wildlife Institute of India, Dehradun; 2011.

[17] Sathyakumar S, Kaul R, Ashraf NVK, Mookerjee A, Menon V. National bear conservation and welfare action plan 2012. India: Ministry of Environment and Forests, Wildlife Institute of India and Wildlife Trust of India; 2012.

[18] Krishnan M. An ecological survey of the large mammals of peninsular India. J Bombay Nat Hist Soc. 1972;69: 47–49.

[19] Seidensticker J, Yoganand K, Johnsingh AJT. Sloth bears living in seasonally dry tropical and moist broadleaf forests and their conservation In. McShae WJ, Davies SJ, editors. The ecology and conservation of seasonally dry forests in Asia. Washington DC: Smithsonian Institution Scholarly Press; 2011 pp. 217–236.

[20] Ratnayeke S, Van Manen FT, Pieris R, Pragash VSJ. Landscape characteristics of sloth bear range in Sri Lanka. Ursus. 2007;18: 189–202.

[21] Servheen C, Herrero S, Peyton B. Bears: status survey and conservation action plan. International Union for the Conservation of Nature and Natural Resources, Gland, Switzerland; 1999.

[22] Bellemain E, Nawaz MA, Valentini A, Swenson JE, Taberlet P. Genetic tracking of the brown bear in northern Pakistan and implications for conservation. Biol Conserv. 2007;134: 537–547.

[23] Dixon JD, Oli MK, Wooten MC, Eason TH, McCown JW, Cunningham MW. Genetic consequences of habitat fragmentation and loss: the case of the Florida black bear (*Ursus americanus floridanus*). Conserv Genet. 2007;8: 455–464.

[24] Paetkau D, Waits LP, Clarkson PL, Craighead L, Vyse E, Ward R, et al Variation in genetic diversity across the range of North American brown bears. Conserv Biol. 1998;12: 418–429.

[25] Tallmon DA, Bellemain E, Swenson JE, Taberlet P. Genetic monitoring of Scandinavian brown bear effective population size and immigration. J Wildl Manag. 2004;68: 960–965.

[26] Paetkau D, Amstrup SC, Born EW, Calvert W, Derocher AE, Garner GW, et al Genetic structure of the world’s polar bear populations. Mol Ecol. 1999;8: 1571–1584. 10583821 10.1046/j.1365-294x.1999.00733.x

[27] Cronin MA, Amstrup SC, Scribner KT. Microsatellite DNA and mitochondrial DNA variation in polar bears (*Ursus maritimus*) from the Beaufort and Chukchi seas, Alaska. Can J Zool. 2006;84: 655–660.

[28] Tammeleht E, Remm J, Korsten M, Davison J, Tumanov I, Saveljev A, et al Genetic structure in large, continuous mammal populations: the example of brown bears in northwestern Eurasia. Mol Ecol. 2010;19: 5359–5370. 10.1111/j.1365-294X.2010.04885.x 21044194

[29] Sharma S, Dutta T, Maldonado JE, Wood TC, Panwar HS, Seidensticker J. Selection of microsatellite loci for genetic monitoring of sloth bears. Ursus. 2013;24: 164–169.

[30] Piggott MP, Bellemain E, Taberlet P, Taylor AC. A multiplex pre-amplification method that significantly improves microsatellite amplification and error rates for faecal DNA in limiting conditions. Conserv Genet. 2004;5: 417–420.

[31] Taberlet P, Griffin S, Goossens B, Questiau S, Manceau V, Escaravage N, et al Reliable genotyping of samples with very low DNA quantities using PCR. Nucleic Acids Res. 1996;24: 3189–3194. 8774899 10.1093/nar/24.16.3189 PMC146079

[32] Van Oosterhout C, Hutchinson WF, Wills DPM, Shipley P. MICRO-CHECKER: software for identifying and correcting genotyping errors in microsatellite data. Mol Ecol Notes. 2004;4: 535–538.

[33] Valière N. GIMLET: a computer program for analysing genetic individual identification data. Mol Ecol Notes. 2002;2: 377–379.

[34] Rousset F. GENEPOP’007: a complete re-implementation of the GENEPOP software for Windows and Linux. Mol Ecol Resour. 2008;8: 103–106. 10.1111/j.1471-8286.2007.01931.x 21585727

[35] Kalinowski ST, Taper ML, Marshall TC. Revising how the computer program CERVUS accommodates genotyping error increases success in paternity assignment. Mol Ecol. 2007;16: 1099–1106. 17305863 10.1111/j.1365-294X.2007.03089.x

[36] Waits LP, Luikart G, Taberlet P. Estimating the probability of identity among genotypes in natural populations: cautions and guidelines. Mol Ecol. 2001;10: 249–256. 11251803 10.1046/j.1365-294x.2001.01185.x

[37] Goudet J. FSTAT (Version 1.2): A computer program to calculate F-statistics. J Hered. 1995;86: 485–486.

[38] Peakall R, Smouse PE. GENALEX 6: genetic analysis in Excel. Population genetic software for teaching and research. Mol Ecol Notes. 2006;6: 288–295. 10.1093/bioinformatics/bts460 PMC3463245 22820204

[39] Lloyd MW, Campbell L, Neel MC. The power to detect recent fragmentation events using genetic differentiation methods. PLOS ONE. 2013;8: e63981 10.1371/journal.pone.0063981 23704965 PMC3660580

[40] Landguth EL, Cushman SA, Schwartz MK, McKelvey KS, Murphy M, Luikart G. Quantifying the lag time to detect barriers in landscape genetics. Mol Ecol. 2010;19: 4179–4191. 10.1111/j.1365-294X.2010.04808.x 20819159

[41] Jombart T. ADEGENET: a R package for the multivariate analysis of genetic markers. Bioinformatics. 2008;24: 1403–1405. 10.1093/bioinformatics/btn129 18397895

[42] Pritchard JK, Stephens M, Donnelly P. Inference of population structure using multilocus genotype data. Genetics. 2000;155: 945–959. 10835412 10.1093/genetics/155.2.945 PMC1461096

[43] Falush D, Wirth T, Linz B, Pritchard JK, Stephens M, Kidd M, et al Traces of human migrations in *Helicobacter pylori* populations. Science. 2003;299: 1582 12624269 10.1126/science.1080857

[44] Evanno G, Regnaut S, Goudet J. Detecting the number of clusters of individuals using the software STRUCTURE: a simulation study. Mol Ecol. 2005;14: 2611–2620. 15969739 10.1111/j.1365-294X.2005.02553.x

[45] Chen C, Durand E, Forbes F, Francois O. Bayesian clustering algorithms ascertaining spatial population structure: a new computer program and a comparison study. Mol Ecol Notes. 2007;7: 747–756.

[46] Bergl RA, Vigilant L. Genetic analysis reveals population structure and recent migration within the highly fragmented range of the Cross River gorilla (*Gorilla gorilla diehli*). Mol Ecol. 2007;16: 501–516. 17257109 10.1111/j.1365-294X.2006.03159.x

[47] Piry S, Alapetite A, Cornuet JM, Paetkau D, Baudouin L, Estoup A. GENECLASS2: a software for genetic assignment and first-generation migrant detection. J Hered. 2004;95: 536–539. 15475402 10.1093/jhered/esh074

[48] Rannala B, Mountain JL. Detecting immigration by using multilocus genotypes. Proc Natl Acad Sci. 1997;94: 9197–9201. 9256459 10.1073/pnas.94.17.9197 PMC23111

[49] Wilson GA, Rannala B. Bayesian inference of recent migration rates using multilocus genotypes. Genetics. 2003;163: 1177–1191. 12663554 10.1093/genetics/163.3.1177 PMC1462502

[50] McRae B, Kavanagh DM. Linkage Mapper connectivity analysis software. Seattle WA: The Nature Conservancy; 2011 Available: http://www.circuitscape.org/linkagemapper.

[51] Rosenberg MS, Anderson CD. PASSaGE: Pattern Analysis, Spatial Statistics and Geographic Exegesis. Version 2. Methods Ecol Evol. 2011;2: 229–232.

[52] Rutledge LY, Holloway JJ, Patterson BR, White BN. An improved field method to obtain DNA for individual identification from wolf scat. J Wildl Manag. 2009;73: 1430–1435.

[53] Broquet T, Ménard N, Petit E. Noninvasive population genetics: a review of sample source, diet, fragment length and microsatellite motif effects on amplification success and genotyping error rates. Conserv Genet. 2007;8: 249–260.

[54] Paetkau D, Strobeck C. Microsatellite analysis of genetic variation in black bear populations. Mol Ecol. 1994;3: 489–495. 7952329 10.1111/j.1365-294x.1994.tb00127.x

[55] Dutta T, Sharma S, Maldonado JE, Wood TC, Panwar HS, Seidensticker J. Fine-scale population genetic structure in a wide-ranging carnivore, the leopard (*Panthera pardus fusca*) in central India. Divers Distrib. 2013;19: 760–771.

[56] Dutta T, Sharma S, Maldonado JE, Wood TC, Panwar HS, Seidensticker J. Gene flow and demographic history of leopards (*Panthera pardus*) in the central Indian highlands. Evol Appl. 2013;6: 949–959. 10.1111/eva.12078 24062803 PMC3779095

[57] Sharma S, Dutta T, Maldonado JE, Wood TC, Panwar HS, Seidensticker J. Spatial genetic analysis reveals high connectivity of tiger (*Panthera tigris*) populations in the Satpura-Maikal landscape of Central India. Ecol Evol. 2013;3: 48–60. 10.1002/ece3.432 PMC3568842 23403813

[58] Sharma S, Dutta T, Maldonado JE, Wood TC, Panwar HS, Seidensticker J. Forest corridors maintain historical gene flow in a tiger metapopulation in the highlands of central India. Proc R Soc B Biol Sci. 2013;280:20131506 10.1098/rspb.2013.1506 23902910 PMC3735263

[59] Crooks KR, Burdett CL, Theobald DM, Rondinini C, Boitani L. Global patterns of fragmentation and connectivity of mammalian carnivore habitat. Philos Trans R Soc B Biol Sci. 2011;366: 2642–2651. 10.1098/rstb.2011.0120 21844043 PMC3140740

[60] Sengupta M. Environmental impacts of mining monitoring, restoration, and control. Boca Raton, FL: CRC Press; 1993.

[61] Jena J, Borah J, Dave C, Vattakaven J. Lifeline for tigers: status and conservation of the Kanha-Pench corridor. New Delhi, India: WWF-India; 2011.

[62] Pragatheesh A, Rajvanshi A. Spatial patterns and factors influencing the mortality of snakes on the national highway-7 along Pench tiger reserve, Madhya Pradesh, India. Oecologia Aust. 2013;17: 20–35.

[63] Forman RT, Alexander LE. Roads and their major ecological effects. Annu Rev Ecol Syst. 1998;29: 207–231.

[64] Riley SPD, Pollinger JP, Sauvajot RM, York EC, Bromley C, Fuller TK, et al A southern California freeway is a physical and social barrier to gene flow in carnivores. Mol Ecol. 2006;15: 1733–1741. 16689893 10.1111/j.1365-294X.2006.02907.x

[65] Kaczensky P, Knauer F, Krze B, Jonozovic M, Adamic M, Gossow H. The impact of high speed, high volume traffic axes on brown bears in Slovenia. Biol Conserv. 2003;111: 191–204.

[66] Beehler BM, Raju KSRK, Ali S. Avian use of man-disturbed forest habitats in the Eastern Ghats, India. Ibis. 1987;129: 197–211.

[67] Areendran G, Sankar K, Pasha K, Qureshi Q. Quantifying land use land cover change in Pench tiger reserve (Madhya Pradesh, India): a landscape approach. Asian J Geoinformatics. 2012; 12.

[68] Balkenhol N, Waits LP. Molecular road ecology: exploring the potential of genetics for investigating transportation impacts on wildlife. Mol Ecol. 2009;18: 4151–4164. 10.1111/j.1365-294X.2009.04322.x 19732335

[69] Kerley LL, Goodrich JM, Miquelle DG, Smirnov EN, Quigley HB, Hornocker MG. Effects of roads and human disturbance on Amur tigers. Conserv Biol. 2002;16: 97–108. 10.1046/j.1523-1739.2002.99290.x 35701953

[70] Quintero JD, Roca R, Morgan A, Mathur A, Xiaoxin S. Smart green infrastructure in tiger range countries: a multi- level approach Discussion papers. Washington, DC: World Bank; 2009 Available: http://documents.worldbank.org/curated/en/ 2010/09/12887165/smart-green-infrastructure-tiger-range-countries-multi-level-approach.

[71] Corlatti L, Hacklaender K, Frey-Roos F. Ability of wildlife overpasses to provide connectivity and prevent genetic isolation. Conserv Biol. 2009;23: 548–556. 10.1111/j.1523-1739.2008.01162.x 19210301

[72] Sawaya MA, Kalinowski ST, Clevenger AP. Genetic connectivity for two bear species at wildlife crossing structures in Banff National Park. Proc R Soc B Biol Sci. 2014;281: 20131705 10.1098/rspb.2013.1705 24552834 PMC4027379

