# Supplementary material for: Genetic Variation, Structure, and Gene Flow in a Sloth Bear (Melursus ursinus) Meta-Population in the Satpura-Maikal Landscape of Central India
Source: PLoS One. 2015 May 6;10(5):e0123384. doi: 10.1371/journal.pone.0123384 (PMC4422521; doi:10.1371/journal.pone.0123384)
Supplement: S1 Fig — (DOCX) [file pone.0123384.s001.docx]

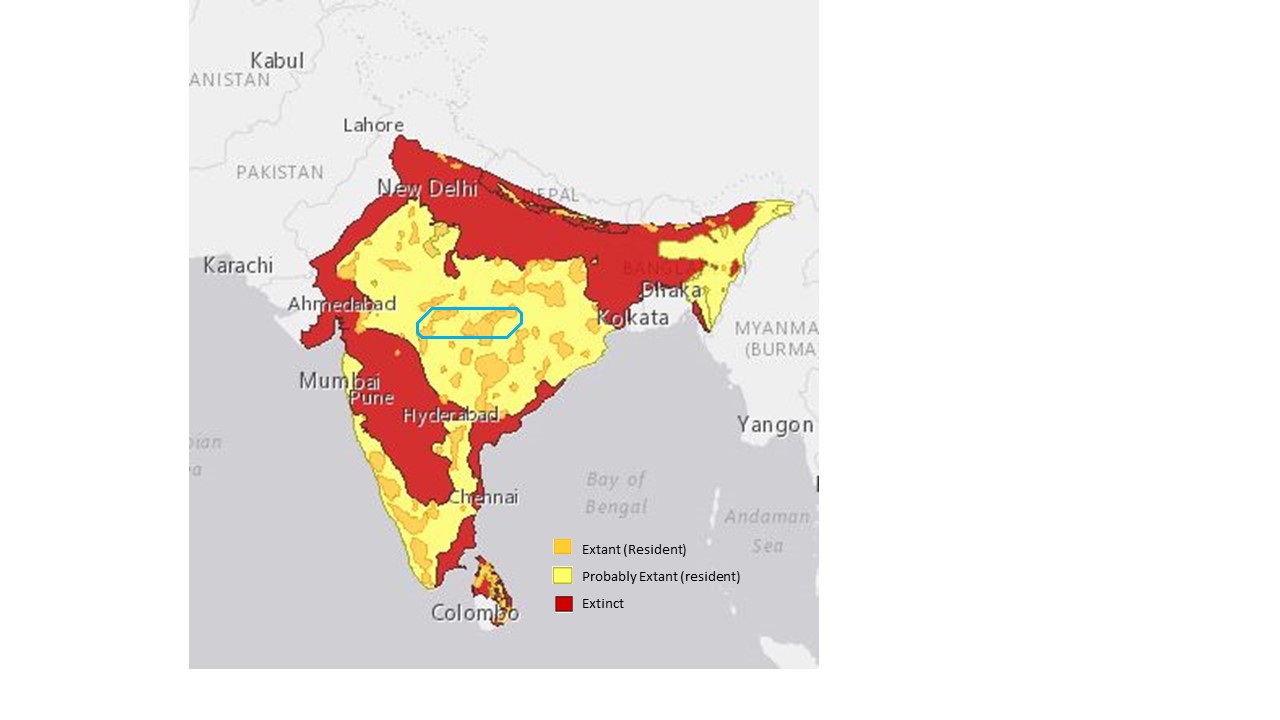
S1 Fig: Range map of Sloth bear (Melursus ursinus) showing the study landscape in the blue box.

Source: Garshelis DL, Ratnayeke S, Chauhan NPS. IUCN SSC Bear Specialist Group 2008. Melursus ursinus. The IUCN Red List of Threatened Species. Version 2014.2. <[www.iucnredlist.org](http://www.iucnredlist.org/)>. Downloaded on 27 October 2014.
