# Supplementary material for: Genetic Variation, Structure, and Gene Flow in a Sloth Bear (Melursus ursinus) Meta-Population in the Satpura-Maikal Landscape of Central India
Source: PLoS One. 2015 May 6;10(5):e0123384. doi: 10.1371/journal.pone.0123384 (PMC4422521; doi:10.1371/journal.pone.0123384)
Supplement: S2 Fig — (DOCX) [file pone.0123384.s002.docx]

S2 Fig: Results from STRUCTURE analysis with fewer samples from Satpura and Melghat.

(a) Δ K plot showing K=2, (b) Barplot from the analysis.

a..

bBarbara Campbell <bcampb7@clemson.edu>.

Kanha

Pench

Satpura

Melghat
