# Supplementary material for: Genetic Variation, Structure, and Gene Flow in a Sloth Bear (Melursus ursinus) Meta-Population in the Satpura-Maikal Landscape of Central India
Source: PLoS One. 2015 May 6;10(5):e0123384. doi: 10.1371/journal.pone.0123384 (PMC4422521; doi:10.1371/journal.pone.0123384)
Supplement: S1 Table — (DOCX) [file pone.0123384.s003.docx]

S1 Table: Characteristics and error rates of genotyping 55 individual sloth bears from Satpura-Maikal landscape in India.

| Locus | Size range | Success | No. of alleles | Ho | He | PID | PID_(SIBS)_ | PID_(cum)_ | PID_(SIBS)cum_ | ADO | FA |
| --- | --- | --- | --- | --- | --- | --- | --- | --- | --- | --- | --- |
| CXX203 | 122-146 | 0.87 | 12 | 0.65 | 0.86 | 2.76E-02 | 3.29E-01 | 2.76E-02 | 3.29E-01 | 0.037 | 0.018 |
| G10B | 133-143 | 0.58 | 4 | 0.27 | 0.47 | 2.92E-01 | 5.91E-01 | 8.06E-03 | 1.94E-01 | 0.067 | 0 |
| G10J | 89-113 | 0.78 | 12 | 0.58 | 0.88 | 1.81E-02 | 3.14E-01 | 1.46E-04 | 6.10E-02 | 0.031 | 0 |
| G10L | 114-130 | 0.96 | 7 | 0.89 | 0.68 | 1.44E-01 | 4.49E-01 | 2.10E-05 | 2.74E-02 | 0.02 | 0 |
| G1A | 173-193 | 0.78 | 11 | 0.38 | 0.72 | 8.70E-02 | 4.16E-01 | 1.83E-06 | 1.14E-02 | 0.063 | 0 |
| UarMu26 | 111-173 | 0.82 | 7 | 0.38 | 0.69 | 1.37E-01 | 4.41E-01 | 2.50E-07 | 5.03E-03 | 0.079 | 0 |
| Umar2 | 185-227 | 0.85 | 9 | 0.58 | 0.71 | 1.05E-01 | 4.26E-01 | 2.61E-08 | 2.15E-03 | 0.063 | 0 |

PID: Probability of identity, PID_(SIBS)_ : Probability of identity among siblings, PID_(cum):_ Cumulative probability of identity, PID_(SIBS)cum_: Cumulative probability of identity among siblings, ADO: Allelic drop out, FA: Proportion of False Alleles.

Source: Sharma S, Dutta T, Maldonado JE, Wood TC, Panwar HS, Seidensticker J Selection of microsatellite loci for genetic monitoring of sloth bears. Ursus. 2013; 24: 164–169. doi:10.2192/URSUS-D-13-00001.1.
